# Update of Probiotics in Human World: A Nonstop Source of Benefactions till the End of Time

**DOI:** 10.3390/microorganisms8121907

**Published:** 2020-11-30

**Authors:** Mohamed Zommiti, Marc G. J. Feuilloley, Nathalie Connil

**Affiliations:** 1 Unité de Protéomique Fonctionnelle et Potentiel Nutraceutique de la Biodiversité de Tunisie, Institut Supérieur des Sciences Biologiques Appliquées de Tunis, Université Tunis El-Manar, Tunis 1006, Tunisia; 2 Laboratoire de Microbiologie Signaux et Microenvironnement (LMSM) EA 4312, Université de Rouen Normandie, Normandie Université, F-27000 Evreux, France; marc.feuilloley@univ-rouen.fr (M.G.J.F.); nathalie.connil@univ-rouen.fr (N.C.)

**Keywords:** beneficial microbes, probiotic, gut microbiota, health benefits, dysbiosis, clinical evidence

## Abstract

Lactic acid bacteria (LAB) are known for their biotechnological potential. Moreover, LAB are distinguished by amazing criteria: Adjusting the intestinal environment, inhibiting pathogenic microbes in the gastrointestinal tract, ability to reduce pathogen adhesion activity, improving the balance of the microbiota inside the intestine, capabilities of regulating intestinal mucosal immunity, and maintaining intestinal barrier function. The escalating number of research and studies about beneficial microorganisms and their impact on promoting health has attracted a big interest in the last decades. Since antiquity, various based fermented products of different kinds have been utilized as potential probiotic products. Nevertheless, the current upsurge in consumers’ interest in bioalternatives has opened new horizons for the probiotic field in terms of research and development. The present review aims at shedding light on the world of probiotics, a continuous story of astonishing success in various fields, in particular, the biomedical sector and pharmaceutical industry, as well as to display the importance of probiotics and their therapeutic potential in purpose to compete for sturdy pathogens and to struggle against diseases and acute infections. Shadows and future trends of probiotics use are also discussed.

## 1. Introduction

Since antiquity, microorganisms have represented, and still are, an indispensable part of human nutrition with high levels of their consumption via naturally microbially fermented products with massive amounts of viable beneficial microbes, such as fermented fruits, their juices, fermented animal products, and other food products of diverse origins. In history, humans, unconsciously, and without even having a clue about the existence of the microbial world, have used microbes in order to initiate numerous food processes [1]. Nowadays, a massive assortment of fermented food products and liquids exists and offers about one-third of global human diets [2]. The human gut microbiota composition plays a key role in homeostasis, balance, gut flora maintenance, and functionality. These two concepts are firmly influenced by several factors, including diet, age, environmental issues, imbalances, ailments, and therapeutic paths. Recently, Gómez-Gallego and Salminen [3] have characterized beneficial microbes, also known as ‘probiotics,’ as efficient modulators of intestinal microbial equilibrium also called homeostasis. The term probiotic is defined with clarity and distinctiveness as ‘live microorganisms that confer a health benefit to the host when administered in adequate amounts’ [4,5,6]. This seems to be the most common conventional definition.

## 2. Probiotics: A bit of History

“It can only be a matter of time, we shall obtain exact information on the influence of diets, which prevent intestinal putrefaction, prolong life and maintain the body’s forces,” Metchnikoff (1907) [7]. Lactic acid bacteria (LAB), particularly beneficial microbes, are referred to as probiotics, terms coined by Nobel Prize winner, Elie Metchnikoff. ‘The indigested food and feces in the intestine are responsible for the production of toxins and shorten human’s life,’ this represented the central hypothesis proposed by Metchnikoff in his masterpiece “The Prolongation of Life,” in which he has elucidated and found the correlation between lactic acid fermented milk regular consumption by Bulgarian and Caucasian people and their health state with a high living average. Tissier [8] and Metchnikoff [7] were the first investigators to set up scientific proposals regarding the probiotic potential of beneficial bacteria. They suggested that the intake of these microbes by patients suffering from diarrhea could restore a healthy gut microflora. Lilly and Stillwell [9] were the pioneers of the term probiotic, describing substances excreted by one microbe to stimulate the growth of another [10]. Fuller [11], for more precision and specificity of the microbial aspect of probiotic microorganisms, reformulated the term as “A live microbial feed supplement which beneficially affects the host animal by 1improving its intestinal balance”. According to Guarner and Schaafsma [12], the most recent but certainly not the last definition of the word probiotics, which has been refined several times, is “live microorganisms, which when consumed in adequate amounts, confer a health effect on the host,” this corresponds to the most adequate probiotic definition (Table 1).

Over the past decades, scientific investigations keen on probiotics and their health benefits have rocketed sky-high. Regardless of the beneficial effects on human health as good bacteria, probiotics have shown high potential in clinical practice. There are reliable proofs that probiotic microbes can hamper various ailments and infections or be useful in their health, particularly in direct connection to numerous gastrointestinal disorders, in both cases, with children and adults [13,14,15]. In the last decades, a wide number of scientific works and studies shed light on the crucial role of probiotic microorganisms and their potential to cure various ailments and disorders based on the promising findings and results of different in vitro and in vivo investigations, suggesting a powerful connection flanked by these so-called probiotics and the human immuno-modulatory responses. Thus, it appears an urgent need for a deep appraisal of several fields where probiotics have been intensely applied, as well as give significant extrapolations in view of novel prospects and future outlooks. That is why literature has tried to report the crucial role played by probiotics and their beneficial effects on different immune disorders, encompassing rheumatoid arthritis, inflammatory bowel disease (IBD), and atopic dermatitis [16]. Other notable health claims include reducing symptoms of lactose intolerance [17,18,19] as well as gut microbiota regulation [20] and digestion improvement [21], antihypertensive [22], host immune system regulation [23], anti-allergic [24], the preventive potential of mammary cancer [25], eczema in children [26], reduction of active ulcerative colitis [27], anti-obesity [28,29], a high decrease of viral-associated pulmonary damage rate [30], and necrotizing enterocolitis in neonates [31]. Moreover, the regulation of several depressive and anxiety disorders has been reported [32,33,34].

## 3. Sources of Probiotic Strains

The main sources may emanate from human origins like human large intestine, small intestine, or even breast milk. It can also be from animal origins, various food biotopes such as raw milk or fermented food products. Probiotic strains isolated from human microflora are well characterized by high adhesive levels to the human intestinal epithelial barrier than others and more likely to be safe. Nevertheless, several probiotic dietary foods and supplements may carry different bacteria and microbes with no history of safe use in humans or in other animals. The bacterial strains used in the supplements and probiotic foods should play a central role in: Cholesterol lowering and its metabolism, colonization in the intestinal, respiratory and urogenital tracts, inhibition of the carcinogenesis, directly or/and indirectly, via the stimulation of the immune system, the metabolism of lactose, the absorption of calcium and the potential of vitamin synthesis, the reductive potential of yeast and vaginal infection, alleviating the rate of constipation and diarrheal disorders, mitigating gastritis and ulcers, helping to reduce acne, rash face and skin problems, and the production of natural antimicrobials. Table 2 presents a synopsis of probiotic microorganisms possibly found in human pharmaceutical and nutritional products.

## 4. Selection Criteria and Requirements for Probiotic Strains

Safety, functionality, and technological utility represent the crucial criteria for the selection of probiotic microbes. This drastic selection was set up according to the World Health Organization (WHO), Food and Drug Organization (FAO), and EFSA (the European Food Safety Authority). Hill and co-workers [6] revealed that probiotic potential is directly connected to particular strains, not to the genus or species of a microorganism. In vitro experiments are available to investigate whether the microbial strains fulfill the above criteria. Based on these selection criteria validated in vitro experiments, it is possible to screen microbes on their potential as probiotic strains. Further tests and validation can be preceded for these microbial strains with animal and human trials. Regarding the safety of a bacterial strain, its origin, its antibiotic resistance profile, and the total absence of virulence determinants associated with pathogenic cultures correspond to the main basis of the safety profile. As for the functional aspects, viability represents a prerequisite for probiotic functionality—it enhances several mechanisms, including adherence to epithelial cells, reduction of mucosal gut permeability, immuno-modulatory effects, and this represents an industrial challenge [38,39].

Lee [40] showed that probiotic strains should answer the requirements, directly linked with the technology of their manufacture, reflecting their ability to survive and uphold their biotechnological properties and potential all through the storage and distribution processes. However, the value of these factors is still under deep conflict as there are different issues of significance, in vitro and in vivo inconsistencies, and lack of standardization to operate procedures. As there is an obvious absence of common standards to the entire probiotic applications, the most relevant approach to establish the properties of a microbial strain is to target populations, in particular, specific physiologic functions via acute investigations [41]. As far as the final product is concerned, the probiotic dose levels should be based on the ones found to be efficient in human clinical trials, and the colony-forming units per gram (CFU/g) of product is a crucial factor. Besides, there is a lack of data about the minimum effective values, it is generally accepted that probiotic products should have a minimum concentration of 10^6^ CFU per milliliter or gram and that a total of some 10^8^ to 10^9^ probiotic microbial amount must be consumed each day for the probiotic benefits to be transferred to the consumer [42].

## 5. Mechanism of Action of Probiotics

In the last two decades, a non-stop advancement has been noticed in the field of investigations on probiotic microorganisms, typically in terms of criteria selection and features of probiotic cultures, their potential use and their direct and/or indirect impact on human health improvement. Inside a human organism, probiotics are responsible for the development of the microflora residing in the gastrointestinal tract (GIT) in the way of ensuring an appropriate microbial balance flanked by pathogens and the good bacteria, also known as homeostasis [43,44]. These beneficial microbes, through this equilibrium, could restore natural microbiota after antibiotic therapy [45,46]. Another astounding role played by probiotics is counteracting the activity of pathogenic intestinal microbiota. Thus, probiotics have a salient potential in inhibiting the growth of sturdy pathogens encompassing *Clostridium perfringens* [47], *Campylobacter jejuni* [48], *Salmonella enteritidis* [49], *Escherichia coli* [50], various species of *Shigella* [51], *Staphylococcus* [52], *Yersinia* [53], *Campylobacter coli* [54,55] and *Listeria* sp. [56,57] thus preventing food poisoning.

An investigation conducted by Vemuri and collaborators [58], showed that the mechanism of action of probiotic microorganisms depends on several factors such as their resistance to colonization, stimulation of phagocytosis, production of antimicrobial compounds, anti-mutagenic effects, chemokines production, and impact on enzyme activity and enzyme delivery. Furthermore, large-scale molecular, bioengineered, and genetic investigations permitted to unravel the basic concept of the beneficial effect of good bacteria so-called ‘probiotics’ with the direct involvement of four mechanisms: (*i*) Microbial antagonism via the exertion of antimicrobial compounds [59]; (*ii*) competitivity with pathogenic bacteria for adhesion to the epithelium and for nutrients [60]; (*iii*) immuno-modulation of the host [61] and (*iv*) inhibition of bacterial toxin production [62].

Recently, there has been an upsurge in the scientific literature directly related to the beneficial effects of probiotic microorganisms on human health and diseases. Various fields of research and investigation in which clinical trials have been published regarding the assessment of the effectiveness of probiotic intake are demonstrated in Table 3 and Table 4. These clinical studies cover certain categories of patients suffering from specific disorders, such as metabolic disorders (diabetes, obesity, dyslipidemia, hypertension), dysbiosis, and gastrointestinal disorders (antibiotic-associated diarrhea, inflammatory bowel disease, constipation, irritable bowel syndrome, diarrhea secondary to treatment for atopic diseases (atopic syndrome and food hypersensitivity, allergic rhinitis), bacterial vaginosis, infections of the respiratory and urinary tract, anxiety and depressive disorders (Table 3).

## 6. Probiotics: A Continuous Source of Health Benefits

There is a broad association between microbiome and human health status, though no claimed causality. In 2018, Cremon and his team [122] reported that overall microbiome shift, dysbiosis, or reduced diversity of microbial communities are in tight connection and/or predispose individuals to ailments and chronic symptoms encompassing inflammatory bowel diseases, irritable bowel syndrome, cancer, allergy, asthma, diabetes, obesity, cardiovascular diseases, neurological and mental disorders, and gastrointestinal imbalances—mainly caused by host genetics and environmental factors such as mode of a neonate delivery, breast-feeding history, diet, infections, use of antibiotics, and other drugs [123,124,125]. Recently, two pilot investigations led by Schmidt et al. [126] and Tao et al. [127] outlined the pivotal role played by lifestyle factors such as smoking, alcohol consumption habit, and physical exercises in direct modulation of gut microbiota by shifting not only its composition but also its functionality.

According to Collins et al. [128], animal models, in vitro experiments, and human clinical trials have shown efficiency and high potential health benefits of probiotics. These beneficial microbes have been reported to restore GIT microflora and provide a wide range of health benefactions (Table 3). Probiotic bacteria have been announced as microbial cell factories of various vitamins, remarkably B group vitamins including biotin, cobalamin, pantothenic acid, pyridoxine, nicotinic acid, thiamine, riboflavin and folate, and vitamin K thus as to nourish the host [129,130]. In the same line, such microbes have been associated with improved nutrient bioavailability and bioconversion, lowered blood cholesterol, alleviated lactose malabsorption symptoms, and reduced incidences of cardiovascular diseases, obesity, diabetes mellitus type II, production of anticancer and antimutagenic substances [131,132]. However, the production of bioactive, immunological, anticancer, and functional compounds are rarely characterized as strain-specific [6]. For instance, the anticancer effect can be achieved by multiple mechanisms: Production of compounds with anticarcinogenic activity, reduction of the activity of enzymes involved in the carcinogen formation, binding and degradation of mutagens, reduction of nephrotoxic and immunosuppressive mycotoxins, inhibition of tumor cell proliferation, and induced cancer cells apoptosis. Likewise, the immunomodulation effect might occur through enhanced production of anti-inflammatory chemokines, immunoglobulins and/or phagocytosis of pathogens [133,134]. Additionally, potential probiotic microorganisms and probiotics have demonstrated an astonishing antagonistic activity toward a wide range of sturdy food and clinical pathogens (Table 4).

## 7. Urogenital Tract Health Care

According to the Centers for Disease Control and Prevention (CDCP), more than one billion women around the world suffer from non-sexually transmitted urogenital infections, such as bacterial vaginosis (BV), urinary tract infection (UTI), and various other yeast infections [146]. In a pilot study conducted by Hanson et al. [147], it has been demonstrated that the most encountered species in direct association with BV are *Gardnerella vaginalis*, *Ureaplasma urealyticum*, and *Mycoplasma hominis*. Sexually transmitted diseases (STDs), recently called sexually transmitted infections (STIs), are also a significant cause of morbidity worldwide. The two most commonly documented bacterial STDs in some developed countries are gonorrhea and Chlamydia, which are caused by *Neisseria gonorrhoeae* and *Chlamydia trachomatis*, respectively [148]. The most critical concern facing the current decade is that despite having sophisticated medicines and therapeutics to treat a wide range of medical conditions, these sturdy pathogens, among others, are concomitantly becoming highly resistant to the present medicines. Therefore, instead of developing new medicines, our present focus should be on developing new live supplements, like beneficial microbes that act against pathogens and confer a lot of benefactions to the organism.

It is pertinent to know that there is a close association between vaginal microbial flora dysbiosis and an increased incidence of urinary tract infection (UTI). There are about 50 different species residing within the vagina, like *Lactobacillus* species encompassing *L. vaginalis*, *L. brevis*, *L. casei*, *L. delbrueckii*, *L. salivarius*, *L. reuteri* and *L. rhamnosus* that are regarded as the salient commanders and regulators of the vaginal microenvironment. Any kind of dysbiosis in terms of vaginal microbial composition greatly influences the health of the vaginal microenvironment, potentially leading to a compromised state of bacterial vaginosis and urinary tract infection. These compromised states can be reassured by balancing the number of *Lactobacillus* sp. via the supplementation of probiotics [146].

## 8. Probiotics Application in Skincare and Cosmetics

According to Huang and Tang [149], cosmetics are defined as substances or products indented to be rubbed, poured, sprinkled, or sprayed on, introduced into, or otherwise applied to the human body or any part thereof for cleansing, beautifying, promoting attractiveness or altering the appearance. The use of probiotics in cosmetic applications has been proposed. Actually, a wide range of probiotics-based cosmetics is used in various forms and formulas, and numerous products are commercially available in the market of cosmetics. However, controlled experimental trials, particularly on humans, are scarce. Diverse patent applications have been reported in this field. Some *in vitro* and animal scientific trials in direct relation to potential cosmetic applications of probiotics have been performed. Marini and Krutmann [150] and Byrd et al. [151] reported the following genera *Staphylococcus* spp. (*St. epidermis*), *Propionibacterium* spp. (*P. acnes*, *P. granulosum* and *P. avidum*) and *Corynebacterium* spp. (*C. simulans*, *C. tuberculostearium*, *C. tenuis*, *C. jeikeium* and *C. xerosis*), as the main bacterial genera in the human skin microbiome. *Staphylococcus aureus*, among a wide collection of sturdy skin pathogens, is responsible for several skin infections, encompassing subcutaneous abscesses, furuncles, impetigo, skin ulcers, and several systemic infections when it reaches into the bloodstream [152,153].

In 2015, Kober and Bowe [154] suggested that orally administered probiotics exert their impact on the skin via diverse mechanisms initiated in the gastrointestinal tract, most likely due to specific immune responses such as modulation of specific T-cells and stimulating toll-like receptors. In the world of the beauty industry, numerous products use probiotics as bioactive ingredients. Among these cosmetic products, soap, lotion, anti-aging serum, wipe, aftershave for topical application, and ingested probiotics drinks are on the top of the list. *Lactobacillus* spp. and *Bifidobacterium* spp. represent the most common probiotics claimed on the label of cosmetic products [149,155]. In the same line, *Lactobacillus* spp. probiotic microbes have been reported to highly manage skin inflammation symptoms, particularly when administered orally. Nevertheless, inconsistent results have been recorded with children in terms of efficacy. Ouwehand et al. [156] reported the therapeutic potential of the commensal skin bacterium, *Staphylococcus epidermis*, of acne-related to *P. acne* and other related skin infections. In accordance with these findings, Cinque et al. [155] found that the bioactive bacterial component in cosmetic formulas, *Streptococcus salivarium* spp. *thermophilus* S244, is able to produce enzymes that significantly reduce skin dryness, loss of tone, and water, thus slowing the process of skin aging. In the same year, Ouwehand and his coworkers revealed, after massive animal and human experimental studies, that the oral administration of *Lactobacillus johnsonii* La1 reduces the effects of ultra-violet radiation (UV) on immune suppression [156]. 

From a body metabolism perspective, Meng et al. [157] reported the astonishing contribution of probiotic microorganisms in the enhancement of exopolysaccharides, antioxidants, bioactive peptides or/and proteins, fermented lyases, and enzymes activity directly associated to modulation of oxidative stress, protecting cells against induced damage by carcinogens and reactive oxygen species (ROS) generated during body metabolism. Cinque et al. [155] conducted a pilot investigation in which an arsenal of antioxidant enzymes involved in the complex skin-antiaging mechanism have been revealed. Amongst these enzymes, glutathione, glutathione reductase, s-transferases, glutathione peroxidases, super-peroxide dismutase, and catalase are the most identified. For instance, the oral intake of lyases enzymes of the probiotic bacterium *Lactobacillus plantarum* K8 led to a significant improvement of forearm and face skin hydration [156]. Sphingomyelinase (SMase), another skin protection enzyme produced by *Streptococcus thermophilus* delivered in exogenous probiotics formulations, was reported to improve skin health and moisture and highly efficient in the treatment of dermatitis and other skin ailments [158]. Furthermore, polyphenols and antioxidants from fruits and vegetables as prebiotics composition play key roles in skincare, skin protection, and antiaging mechanisms, providing synergetic effects on probiotics [156,159].

## 9. Angiogenic Activity of Probiotics

According to Folkman [160], angiogenesis represents a crucial phenomenon and essential for the wound healing process through delineated cellular responses to regenerate damaged tissues. The angiogenic system consists of a deliberately orchestrated series of cellular events by which new vessels arise from preexisting ones by promoting the recruitment of inflammatory cells and producing cytokines, matrix-degrading enzymes, and chemokines. Deregulated angiogenesis has a salient impact on major human diseases, encompassing cancer, diabetic retinopathy, and irritable bowel disease (IBD), including Crohn’s Disease (CD) and ulcerative colitis (UC) [161,162].

*Saccharomyces boulardii*, a nonpathogenic probiotic yeast, has been announced with protective potential against intestinal injury and inflammation. The molecular mechanisms by which probiotics intercede these benefactions are by all means so intricate and remain blurred. The potential mode of action of probiotics in the angiogenesis process may include alteration of inflammatory cytokine profiles, down-regulation of pro-inflammatory cascades or induction of regulatory mechanisms also known as the feedback circuit, in a strain-specific manner, epithelial barrier function improvement, visceral hypersensitivity reduction, spinal afferent traffic, and stress response.

## 10. Fecal Microbiota Transplantation

Fecal microbiota transplantation (FMT) represents a revolutionary technique in the microbial therapeutic field. This therapy is prompt engraftment or infusion of fecal suspension of a healthy donor into the gastrointestinal tract of an ailing subject for the purpose of curing a specific disorder in direct connection with gut microflora dysbiosis and confer a health benefit [163,164]. FMT is also used in various gastrointestinal disorders, encompassing irritable bowel syndrome (IBS), inflammatory bowel disease (IBD), and idiopathic constipation [165]. The beneficial health claims of FMT in metabolic syndromes, neuro-developmental disorders, allergic complications, and autoimmune diseases were also reported [165,166]. Historically, Ge Hong was the first who described the use of feces as therapy in the fourth-century and reported in China for the treatment of a variety of conditions, including severe diarrhea [167]. A pilot study performed by Eiseman and co-workers [168], in which the use of fecal enemas as a treatment for pseudomembranous colitis has been demonstrated, and this notably marked the introduction of FMT into mainstream medicine. This approach has gained attention with numerous clinical studies published [165]. A large amount of scientific literature, counting ‘RCTs’ referring to Randomised Controlled Trials, meta-analyses studies, and systematic reviews established obvious proofs that fecal microbiota transplantation is a highly successful and efficient remedy against *Clostridium difficile* infection, particularly recurrent *Cl*. *difficile* infection (rCDI) [169,170,171,172]. Due to the non-stop growing occurrence, sternness, and mortality of this severe infection, the curative potential ensured by FMT is, without question, a salient tool to save human sanitary state and life and to reduce the economic and financial burden on healthcare systems [173,174,175]. For instance, Vandenplas et al. [176] have reported for the first time an entire cure of early-onset colitis after FMT approach application. It is also reported that FMT successfully inhibited the multi-drug resistant gut pathogens in an immunocompromised patient [177].

However, according to Rubin [178], results are often negative, and there are quite a few risks associated with FMT. Some adverse event, such as bacteremia has been reported [179]. That is why the transplanted microbiota should be carefully screened for pathogens [180]. Thus, the impact of FMT on the intestinal microbiota and immune system is erratic, and there is still an urgent requirement of large-scale and well-designed quality randomized clinical trials (with different pathways of administration) to acutely define the role of FMT as a proper intervention. Currently, further investigations must shed light on the main reasons for failure, success, and potential use.

## 11. Human Gut Microbiota: “A Second Brain”

The microbial world is able to colonize every single inch of the human body’s surface directly exposed to the environment, with most residing microbial communities in the gastrointestinal tract (GIT). The human GIT is one of the most complex ecosystems known. It harbors one of the most miscellaneous and rich bio-niches on earth colonized by more than 100 trillion (10^14^) microorganisms [181,182]. This tremendous number is imposing in comparison with the entire human cells in a single individual, counting about 10 trillion (10^13^) cells. The human gut contains a diverse and complex microbial community, which plays a crucial role in human health. The human gut microflora holds an estimated amount of 1.5 kg of microbes [183]. This mass of bacteria is termed microbiota, and by adulthood, it is stable in terms of genera but will vary in terms of species between individuals [183]. A strong correlation has been noticed between bacterial composition and their distribution with the nutrient requirements [184]. Numerous trials and researches have investigated the composition of the human gut microbiota [185,186,187,188,189,190]. It has been shown that it is composed of an astonishing microbial diversity with more than one thousand symbiotic and/or commensal microbial species among which we could find Archaea, Bacteria, Viruses (including bacteriophages), even unicellular eukaryotes have been found, including Fungal species and other microbial communities encompassing non-archaeal and non-bacterial microbial species. The bacterial component of the gut microflora and its central role in human well-being has been inspirational for the instigation of large-scale elite projects such as the ‘HMP’ referring to Human Microbiome Project, driven by Turnbaugh and co-workers [183] and Metagenomics of the Human Intestinal Tract (MetaHIT) project led by Qin and collaborators [191]. The non-stop evolution and advances of molecular techniques and metagenomics, in particular genomic sequencing, have shown that the collective adult human gastrointestinal tract microbiota is composed of up to 1000–1150 bacterial species exceeding 10 times or more the total number of host cells [191,192,193,194]. These elite projects and other connected investigations have generated a wealth of data, therefore, giving a more detailed description of the human intestinal microflora composition and of its physiological and metabolic processes and function [195,196], suggesting a strong connection flanked by the gut microflora and their genomic potential in the preservation of general human well-being [197], and its role in maintaining highly particular body functions including development of the immune system [198], neuro-developmental ailments [199], and xenobiotic metabolism [200]. 

## 12. Probiotics: Do These Microbes Confer any Benefits for Generally Healthy People?

Probiotic intervention investigations have been performed on healthy or vulnerable subjects, also known as ‘at-risk’ subjects targeting diverse clinical endpoints. Taken jointly, these experimental studies put forward that there may be some benefit in free-living, generally healthy people. For instance, experimental studies have shown that probiotic bacteria can modestly decrease the incidence [201] and duration [202] of common upper respiratory tract infections in children. According to Wang et al. [203], some evidence exists for certain probiotics to help manage blood lipids in people with mild hypercholesterolemia. Similarly, Oak and Jha, [204] showed that beneficial microbes are able to improve lactose digestion in lactose-intolerant people, with heavy evidence showing that lactose consumed in yogurt with live cultures is better tolerated than the same amount of lactose consumed without live cultures. In this field, an excellent systematic review has been written in order to assess the role of probiotics in the management of lower gastrointestinal symptoms [205]. The included experimental studies and clinical trials covered a wide range of clinical symptoms experienced by healthy subjects and/or subjects with slight gastrointestinal symptoms. Consensus statements were considered by a panel of 14 experts on behalf of the European Society for Primary Care Gastroenterology. In brief, the panel agreed that specific probiotics should be tried or considered for the management of several symptoms of Irritable Bowel Syndrome (IBS), although not all probiotics showed benefit. Observed benefits for IBS may be of modest scale, strain-specific, and depend on the host physiology, diet, and colonizing microflora.

## 13. Current Challenges in Lactic Acid Bacteria Application as Probiotics

The application of probiotic LABs has been mitigated by certain face-to-face challenges. Although the astonishing wave of benefactions of probiotic microbes, these claims can only be asserted if a high number of viable cells reach the small intestine. Various probiotic bacteria have been shown vulnerable and unable to resist long-term exposure to low pH after fermentation and/or oxygen during refrigeration distribution and storage of products and the acidic conditions in the human stomach [206,207,208]. In the same line, it is pertinent to know that the viability and survival of probiotic bacteria are strain-specific criteria. Thus, microencapsulation techniques such as solvent evaporation have been successfully applied to protect the bacterial cells from physicochemical damages caused by environmental biotic and abiotic factors. In a preliminary microencapsulation investigation, Evivie [208] reported that more viable *L*. *plantarum* cells were obtained than *B. breve* cells. Amongst challenges, we can find organoleptic and sensory acceptance of probiotic food products. For instance, various studies have reported the possibility of obtaining similar, or even better, performance with probiotic products as compared to conventional food products such as: Functional yogurt supplemented with *L. reuteri* RC-14 and *L. rhamnosus* GR-1 [209], chocolate mousse with added inulin and *L. paracasei* [210], curdled milk with inulin, and *L. acidophilus* [211], and milk fermented with *B. animalis* and *L. acidophilus* La-5, and supplemented with inulin [212]. Another wave of challenges has been reported by Champagne et al. [213] and Antoine [214]. It encompasses inoculation, appraisal of the viable counts of the probiotic strains in particular, when multiple probiotic bacterial strains are added, and when there are also bacterial starter cultures added. It also includes maintaining probiotics, diversity, and origin of beneficial microbes, probiotic survival, and their active state, dealing with endogenous microflora, intrinsic intricate factors, and proving health benefits.

In a nutshell, more investigations must be performed in terms of studying the interaction between probiotics—other microbes—food matrices axis despite the detailed studies of the physiology of lactic acid bacteria. These include a cascade of mathematical models and approaches investigating LAB bioinformatics, which can be used to sensibly predict responses of these microbes in certain food matrices as well as explore further applications. According to Dos Santos et al. [215], the upsurge of omics and next-generation sequencing tools is gradually making the picture less hazy, but there is still much to be done. Recently, Landete [216] opined that while genetic engineering of LABs can have several positive endpoints and outcomes on the food and pharmaceutical industries, it could be limited by legal issues and safety regulation surrounding the use of the technology, which is still controversial in some quarters. This has, in part, contributed in fewer scientific works investigating the use of GRAS recombinant LAB strains a.k.a Genetically Modified LABs (GM-LABs) that lower the onset of obesity and T2D biomarkers. It is hoped that when some of these hurdles are addressed, more biotechnological and related applications should be in the near and/or far horizon.

## 14. Safety of Probiotics

According to Anadón and co-workers [217], the appraisal of probiotics’ safety represents a delicate task. Generally, probiotic microorganisms are well distinguished by their safe aspect with GRAS status (Generally Regarded as Safe) by the World Health Organization (WHO) [218]. Safety for human health corresponds to the salient determinant for probiotics selection. According to Snydman [219], probiotic strains should be characterized by the absence of their virulent profile and their low resistance to antibiotics. These beneficial microbes have a good safety record during history, primarily related to the use of *Lactobacilli* and *Bifidobacteria* strains [220]. Experience and field trials with other microbial species used as probiotics are more limited. From a standard point of host susceptibility, there is no such thing as zero risk [220]. The selective criteria of new potential probiotic microorganisms target new bacterial strains and even new genera with higher beneficial potential and/or with more particular properties, and this is not an easy task. The introduction of novel microbes needs acute investigations and assessment of their safety and the risk-to-benefit ratio. New probiotic bacteria must belong to genera and strains commonly found in the healthy human intestinal microbiota, and caution must be taken for bacteria belonging to the genus *Bacillus* or *Enterococcus,* in which pathogens or opportunistic pathogens have also been described [221].

The majority of probiotics are safe. Nevertheless, adverse effects have been sporadically reported, and caution of potential side effects should be taken. In 2002, a report jointly released by the World Health Organization (WHO) and the Food and Agriculture Organization (FAO) (http://www.fda.gov/ohrms/dockets/dockets/95s0316/95s-0316-rpt0282-tab-03-ref-19-joint-faowho-vol219.pdf), in which they proclaimed that probiotics may, at a theoretical level, be in charge of four types of collateral effects”: (*i*) Systemic infections; (*ii*) deleterious metabolic activities; (*iii*) excessive immune stimulation in susceptible individuals, and (*vi*) potential gene transfer [4].

In fact, several cases of side effects have been reported, such as rare incidents of sepsis, endocarditis, and liver abscess during the use of *Lactobacillus*. In addition, fungemia cases have been reported with the use of *Saccharomyces boulardii*, primarily in patients with severe comorbidities [219,222]. Constipation, flatulence, nausea, hiccups, infection, and rash represent the most common drawbacks of probiotic use. Besselink and collaborators [223] reported that probiotics consumption by acute pancreatitis subjects has no effect on infection complications and instead increases the mortality rate. Several *Lactobacilli* bacteremia have also been reported [224]. According to Allen et al. [225], the administration of probiotics during pregnancy and early infancy is considered safe and not related to side effects. In a standard point of view of systemic infection, broad epidemiological investigations in different countries, where probiotic use is prevalent, revealed (in adults) low levels of systemic infection, flanked by 0.05% and 0.40%, respectively [226]. According to Fedorak and Madsen [226], the public understanding of the idea of risk and risk/benefit seems weak and poor. Uncertainty concerning the ability of antibiotic resistance transfer with probiotics is an actual issue to think about, though the risk seems to be low with probiotic products currently available in the markets. Regarding other various forms of therapeutic agents, probiotics and their biosafety must be assessed on a strain-by-strain basis [226].

To sum up, the world of probiotics is continuously growing, not only by the increasing number of people who use probiotics but also by the variety of probiotic products and novel probiotic strains. Future investigations and scientific studies need to report a more detailed description of the tested probiotic microbe encompassing the genus, species, and strain level, additionally to the daily dose and the duration of the treatment [227,228]. Three major elements composed from the public, healthcare providers and manufacturers have to win the challenges face to face to probiotics, in purpose to focus on international regulations and standards and to provide guidance for strain-specific evidence-based therapy.

## 15. Regulation of Probiotics Safety around the Globe

The upsurge in the world of probiotics in terms of their use has been accompanied by the absence of a universal standard for safety assessment and regulation, reflecting a high variation from country to country or regional wise [229,230]. Around the globe and entirely dissimilar to commercial drugs, probiotics are mostly categorized as food and/or dietary supplements in the United States of America and Europe, as natural health products in Canada, and as food for specific health use in Japan. This categorization fulfills with significantly less drastic regulations [230]. For instance, in the United States of America (U.S.A.), the GRAS status (Generally Recognized as Safe) represents a salient criterion for bacterial safety. A classification provided by the Food and Drug Administration (FDA) and such products is not subjected to drastic monitoring [231]. Health Canada accepts 1 × 10^9^ CFU (Colony Forming Unit) per serving for nonspecific claims as probiotics, which might comprise the matrix of *Bifidobacterium* and *Lactobacillus* spp. in the product containing the probiotic formula [6].

The European Food Safety Authority (EFSA), inside the European Union, suggested an introduction on the QPS status (Qualified Presumption of Safety) that could be applicable to select groups of beneficial microbes for direct human consumption [232]. The QPS status is based on taxonomy, characterization, pathogenicity, and final statistical data for conformity in order to qualify a probiotic. Additionally, the EFSA agency is the first responsible for the assessment of the probiotics health claim made on food that is presented by various manufacturers [232]. For instance, the Italian ministry of health controls the use of probiotic bacteria in food products and set the permissible minimum number of viable cells in food per serving at 1 × 10^9^ CFU per day [6].

In China, the Chinese State Food and Drug Administration (SFDA) regulates and supervises functional foods and nutraceuticals [233,234]. Probiotics, amongst functional food products, take a large market share in China, highly influenced by traditional dietary culture and habits along with economic growth.

In Japan, probiotics are regulated under the Food for Specific Health Use agency (FOSHU), which authorizes labeling with health claims on food or ingredients that meet scientific evidence required for safety and efficacy [235]. According to Foligné et al. [233], the FOSHU products are endorsed by the Japanese Minister of Health, Labor, and Welfare. The claims are classified as: Special dietary uses, specific health applications, and food with nutrient function [236]. The health traits may cover diverse features in terms of cholesterol, triglycerides, blood pressure, blood sugar, bone minerals, and dental health. Moreover, the appraisal of any sort of innovation is based on the source and the traditional use of foods and/or food ingredients.

According to Cremon and coworkers [122], the FAO/WHO recommended that the probiotic products should be labeled in terms of various details and information encompassing genus, species, and strain designation, minimum viable number of each probiotic strain at the end of the shelf life, storage conditions, and traceability information such as cooperate contact. Although, regulatory standards on probiotics are not established on an international basis, reflecting a critical absence of periodic screenings of the product’s safety and quality. While products added with probiotics are not strictly legalized, an outstanding review by Kolaček et al. [230] presented some aspects of the quality evaluation from the world all over, including U.S.A., Europe, Asia, South Africa, and Australia with critical alarm on misinterpretation at the genus, species, and strain level and mislabeling. In two pilot investigations performed in 2014 and 2017 by Chen and collaborators, it has been reported that the claimed number of viable bacterial cells per dose of many probiotic food products are significantly inferior to those on their labels [237,238]. Microbial contamination and lower techno-functional properties of probiotics, which are influenced by processing, handling, and food matrixes, represent major challenges that probiotic application may face.

According to Zoumpopoulou et al. [239], several isolated cases of side effects of probiotics, in terms of transmission of antibiotic resistance genetic determinants to pathogens and production of biogenic amine, have been reported. Therefore, the following information is recommended to be revealed: Isolation history and/or origin, taxonomic identification, absence of virulence, toxicity, and chances of antibiotic resistance gene transfer to pathogenic bacteria. Notably, EFSA and GRS in 2007 and 2012, respectively, introduced antimicrobial resistance as the safety concern on probiotics consumption due to horizontal gene transfer from beneficial to pathogenic bacteria in the GIT [240].

## 16. General Discussion: Probiotics and a Glance on Tomorrow

A salient concept is that there can be no sense of global security without first ensuring food security. This comprises, amongst other factors, the stipulation of safe and nutritious food for the globe’s teeming population. Given the enormous opportunities that exist in the use of LAB as probiotic microorganisms, the future is indeed shiny and promising in terms of safety and security. For instance, one crucial arena that is currently being tapped into is investigating probiotic propensities and tendencies through complete genome sequencing technology. This, among other effects, will boost the techno-functional properties of probiotic LAB, and data thereof can be used as a good basis to further manipulate LAB genes [241]. More investigation into their use as functional food components is currently in progress and is expected to increase in the nearest future. Recently, an entire nonstop propensity in terms of researches and investigations into the attenuating effects of probiotic LAB on breast cancer cells and the associated types of carcinomas, thus further bridging the gap between the food, health, and medicine fields of the world [242]. However, it must be stressed that improving food safety and security is not an easy task, it is not a probiotic characteristic of lactic acid bacteria only, it is about an intricate network of various factors.

In the last few years, an outstanding upsurge of studies investigating the molecular basis for the potential probiotic properties of prospective LAB strains and their products have emerged, radically improving our understanding of their biology [243,244,245,246]. These reports have formed and still form the basis for *in vitro* and *in vivo* studies, which will be of paramount importance to experts in the fields of food, biomedical, and pharmaceutical industries. For instance, the Key Laboratory of Dairy Science (KLDS) of the Northeast Agricultural University (NEAU), China, have reported that a recently sequenced lactic acid bacterium strain, *Streptococcus thermophilus* KLDS 3.1003 (GenBank Accession Number: CP016877) and its cell-free supernatant (CFS) can have an astonishing antagonistic activity against food-borne and vaginal pathogens (*Staphylococcus aureus*, *Escherichia coli,* and *Gardnerella vaginalis*) (unpublished data). Such exciting findings can, amongst others, be integral in the development of useful and paramount components in the development of novel functional foods, production of bio-drugs as well as bio-vaccines. 

Amensalism corresponds to a way of one microbe gaining an advantage over another competing bacterium. This can be accomplished via the modification of the biotope through its acidification and releasing antimicrobial substances and compounds inhibiting and/or even killing the growth of competitor bacterial populations. Antimicrobial compounds and metabolites are actually produced by bacterial species as byproducts of metabolism. These bacteria are able to produce a sophisticated arsenal of antimicrobial bio-weapons encoded by genetic determinants designed specifically to combat other undesirable pathogens. LAB solo or associated with their antimicrobial compounds and peptides have received a considerable interest in recent years as food biopreservatives. Based on the wide spectrum of their antimicrobial compounds, LAB can be exploited as microbial cell factories of natural antimicrobials for food biocontrol [247]. The full characterization of antimicrobial substances produced by probiotic LAB is also becoming a central area of investigation as a result of their increasingly important industrial, nutritional, and therapeutic applications. In this regard, a closer examination of probiotic LAB over the past decades has revealed its potential to produce an arsenal of antimicrobial substances of different structures [248,249]. The main substances comprise organic acids, biosurfactants, exopolysaccharides (EPS), and bacteriocins. Till now, most scientific studies, for several limiting factors, have only been able to partially characterize these antimicrobial substances, but this has to be urgently improved on using new tools and approaches thus as to open new horizons. Some pertinent questions have been asked, such as can probiotic LAB be used to develop anti-inflammatory yogurts, cholesterol-lowering cheese, anti-diabetic ice creams, desserts, and the likes as earlier postulated? Can appealing scientific discoveries about the structure of these beneficial microbes give new insights into their potential use in treating ailments of viral origin? Will more in vivo studies emerge, showing more direct correlations between the immunomodulatory activities of LAB and their suppressing the onset of predisposing disease factors? The nearest sunrise will hopefully answer these and even many more.

Additionally, research into the genetic components, genotyping, and genetic profile of probiotic LAB strains can also have outstanding advantages in eliminating incidences of food poisoning. This latter critical issue actually accounts for an estimated 420,000 deaths/year, which is a deleterious burden to the economy of any nation [250]. This is rightly thus as food is undoubtedly an important vector not only for nutrients but also for the favorable development of sturdy pathogens. Given the growing interests in ensuring that foods consumed daily are both nutritious and safe [251,252], understanding how the genes of promising probiotic bacterial strains suppress the gene expression and accordingly the survival of notorious reputed food-borne pathogens and their toxins may open new horizons and shed light on the salient potentialities of lactic acid bacteria as probiotics. Numerous studies [208,253,254] have worked on synbiotics and the application of microencapsulation as a novel approach in order to ensure the survival of more viable probiotic LAB cells for significant health impact. This will be of particular significance to low- and middle-income countries, which are most affected by food-borne diseases [245]. It is pertinent to know that food safety is a shared responsibility. It must also be emphasized that consumer education be highly intensified to guarantee that healthy food choices are constantly and consciously made as this is requisite for a healthy lifestyle and living. The nearest future may also see the appearance of a bouquet of more probiotic, prebiotic, and symbiotic ice-cream products, which may serve a variety of uses. The technological and non-technological downsides must first be tackled before desired significant advances can be made. 

Based on the experimental trials and clinical studies presented in this review, it is, therefore, hypothetically feasible that multiple bacterial strain combinations be used to investigate the reducing potential of probiotic bacteria and *Bifidobacterium* spp. on gut microflora in terms of attenuating diverse disorders and ailments. Such studies may unveil new and novel pipelines for bio-drugs and new functional foods and food products with colossal industrial applications. Recent investigations on the subject can serve as a strong theoretical platform for further researches into the modulation of the gut microbiota via testing the effects of single or multiple probiotic LAB strain dosages [255,256]. In the same line, a pilot study conducted by Panwar and collaborators [257] in which it has been reported that although several *Lactobacillus* strains showed strong DPP-4 inhibitory activity, *Escherichia coli* 0157 and *Salmonella typhimurium* showed the strongest inhibition (30–32%). Investigations into the manipulation of these pathogenic microorganisms for probiotic purposes may thus be needful for future research. The nearest future may also see the emergence of novel starter cultures and probiotic bacteria with tremendous biomedical and therapeutical potentialities with more *in vivo* trials to support the claim that probiotic lactic acid bacteria may indeed have more direct effects on the suppression of pathways and processes within the human GIT that predisposes people to obesity, diabetes, cancer, and other metabolic and non-metabolic disorders. It is hypothesized that this line of investigation will greatly lower the current ethical, cultural, or religious barriers impeding lactic acid biotechnology research and promotion of functional food ingredients.

In this work, there is a brief knowledge of applications and potential applications of diverse probiotic bacterial strains in human health. The efficacy of probiotic microorganisms could be boosted by using the mixture of probiotics and prebiotics. For this purpose, *Bifidobacteria* can act as a valuable adjuvant for the improvement of techno-functional properties of a probiotic.

A person acquires his/her microbiota at the time of birth from his/her mother’s health as well as from surroundings. The presence of good microflora leads to good health conditions. Extrinsic factors such as foreign microorganisms, ailments, and massive use of antibiotics may lead to disturbance of gut microflora. This latter may be restored, in a case of dysbiosis, imparting various benefactions to humans reflecting the salient commander of the human health. Probiotic microorganisms come from different sources encompassing fermented foods and fermented food products, animal origin, and even human origin. Recently, there is an urgent requirement of designing new and improved forms of probiotics for their applications in the field of food and health intended for livestock and humans.

Scientific investigation suggests that probiotics can be used in an evidence-based manner to address a range of different health concerns. For the healthy subject, probiotic microbes and fermented foods may provide a dietary approach to support health and better function of the gut microbiota. The potential roles of probiotics in reducing disorders associated with faulty immune programming leading to autoimmune disorders or correcting dysbiosis that might influence metabolic diseases and issues of stress, anxiety, depression, and other expressions of brain function are also being investigated.

Research is needed to determine if current probiotics or the new buzzword next-generation probiotics (NGPs) can help address these various disorders and ailments. This new buzzword has a heavy impact on human dietary and biotherapeutical portfolios, which are currently the major focus of attention worldwide. Next-generation probiotics respond to the requirements of the normal definition of a probiotic. Besides, we are, above all, designing those microorganisms that have not been used as agents for health improvement, and which are more likely to be delivered under a drug regulatory framework. Among these microorganisms, we could find *Bacteroides xylanisolvens, Bacteroides fragilis*, *Clostridium butyricum*. Next-generation probiotics also fit well within the US Food and Drug Administration definition of live biotherapeutic products: “A live biotherapeutic product that: (*i*) Contains live organisms, such as bacteria; (*ii*) is applicable to the prevention, treatment, or cure of an ailment or condition of human beings; and (*iii*) is not a vaccine”.

Regarding the effects of probiotics, these latters could be highly enhanced by the development of nano-encapsulated probiotics (the shelf life of the product can be improved via encapsulation) by using nanotechnology applications. In the same line, the World Health Organization (WHO) suggested that probiotics will be the salient tool to fight against a wide range of infectious and noninfectious diseases in place of antibiotics, which show many devastating effects in addition to antibiotic resistance and the emergence of highly resistant sturdy pathogens. Antibiotic resistance, as a heavy burden on the public health system, can be treated by probiotic application, also known as microbial interference therapy. Thus, that time is not so far when probiotic microorganisms will enclose the world of medicines and biotherapeutics in the medical and/or pharmaceutical fields. Harmoniously with this propensity, recent advancement in biotechnology helps to isolate and colonize microorganisms to determine their specific biotherapeutic properties and uses. In countries like Japan, Europe, and Australia, probiotics and their related products currently occupy the largest sector in the food market, reflecting their relevant importance. The European Commission has sponsored research projects for the safety and efficacy of the products. 

To wrap up, it is pertinent to know that actual clinical and nutritional assessments have been successfully crowned by exposing some astonishing functions and properties of particular probiotic strains. Specifically, regulation of energy in various catabolic and anabolic processes, acid and bile tolerance, ability to adhere to gut epithelial cells, to fight against sturdy pathogenic bacteria, along with certain other properties, like their safety-enhancing property, serviceability as food, and beneficial supplements for human health. Consequently, the current focus is on evaluating new strains of probiotics and their applicability in biomedical, pharmaceutical, and clinical research, paving a new direction for exploration and exploitation of probiotics aimed at improving human health.

## 17. Concluding Remarks and Future Perspectives

An outstanding review has been written by Zendeboodi et al. [258], in which the term ‘probiotic’ has been drastically revised and conceptualized. For instance, over decades, the definition of the specific term has been changed and broadened. Although the definition developed by WHO/FAO is widely accepted by the majority of investigators, recently, new definitions have been added to the probiotic terminology such as parabiotics and postbiotics. The divergence in terms of the definition of probiotics led to an imperative new approach and conceptualization in probiotic terminology to be developed for global usage in all scientific literature.

Without ensuring the concept of food security, there can be no such meaning of global safety. This may encompass, amongst many other features, the stipulation of safe and nutritious food for the swarming population around the globe. For years, the use of LAB as probiotics has shown its efficiency and great beneficial impact on animal, but especially on human health, reflecting, without question, a promising future of this field. Currently, one area is being tapped into is investigating probiotic tendencies via whole genome sequencing technology. This, amongst other features, will boost the functional aspects of probiotic lactic acid bacteria and data will provide a good base for further LAB genes’ manipulation [259]. The gastrointestinal microbiota is essential to ensure a healthy immune system’s development. Though, the salient indications of the use of probiotics in the medical field are still limited in the zone of prevention and/or treatment of gastrointestinal-associated diseases, more evidence needs to be gathered on extra-intestinal signs, including atopic dermatitis, respiratory, and urogenital tract infections. Probiotics and struggling against cancer with its various types represent an interesting field for research and studies. For instance, ensuring a strong correlation linking three central sectors, including the food, health, and medicine around the globe, is a major key to the pursuit of this battle [242]. It is important to know that food safety represents a shared responsibility to ensure sustainability. This critical issue is strongly influenced by consumer education, which is highly intensified in purpose to ensure a close link between healthy food choices on the one hand and healthy life and living on the other hand. Currently, *Lactobacillus rhamnosus* GG and *Saccharomyces boulardii* represent the most and the best-studied strains, whilst current scientific research supplies positive data on *L. reuteri*. A new tendency that may soon, probably within the next 5 years, be a reality, is the development of new biotherapeutic strategies, encompassing the development of phagebiotics, psychobiotics, and (genetically modified) pharmabiotics [260,261,262]. Misuse and use of products not yet validated may constitute potential drawbacks, which should be tackled before further significant advances can be made. 

In this field, it is relevant to note that besides the beneficial effects of probiotics, these latters may have collateral effects if not appropriately used. Receiving probiotics in high-risk populations posed some health complications reflecting a critical burden in the public health sector. Along with the genetic characteristics of intestinal microbiota in each human case, probiotics and their impacts on adults are being influenced by diverse factors counting environmental factors, diet, and use of antibiotics. Thus, viewing non-identical outcomes in different age groups of consumers would be predictable events, and consequently, precaution is mandatory to be taken just prior to receiving probiotics. Current investigations are under intricacies due to some restrictions in various factors encompassing the lifestyle of case studies, dissimilarity in the normal microflora of humans, gene relevant differences, sex, age of evaluated subjects, and the difference in the period of treatments or follow-up times that all could lead to dissimilar findings and a variety of success reports. From a standard point of view, the best probiotics in terms of dose, duration of treatment, and efficiency require further scrutiny and cannot be underplayed. Our recommendation for the researchers is to focus on the effects of probiotics sources to the host and food and/or clinical safety or adverse effects of probiotics in high-risk consumers.

## Figures and Tables

**Table 1:** History of probiotics—discoveries and highlights.

Period	Discoveries and Highlights
1857–1864	Pasteur discovered LAB as spoilage organisms
1878	LAB isolated from milk by Lister
1889	Tissier described *Bifidobacterium*
1907	Metchnikoff describes Bulgarian *Bacillus* associated with health
1900	*Bacillus acidophilus* described by Moro
1930	The commercialization of fermented milk-based on *Lactobacillus casei* isolate by Shirota
1953	The use of the term ‘probiotika’ referring to active compounds promoting health
1965	Definition of probiotics by Lilly and Stillwell: “Microbes stimulating growth of other microorganisms”
1989	Definition of probiotics by Fuller: “Beneficial microbial supplements”
2001	FAO/WHO: Definition of probiotics
2003	Era of Genomics: First genome sequencing of the probiotic *Lactobacillus plantarum*
2005	Relman and the use of high-throughput 16S amplicon sequencing to catalogue gut microbiome
2016	FDA/CBER guidelines for live biotherapeutics

FAO: Food and Agriculture Organization; WHO: World Health Organization; FDA: Food and Drug Administration; CBER: Center for Biologics Evaluation and Research.

**Table 2:** Probiotic microorganisms used in the human pharmaceutical and nutrition field [35,36,37].

Microorganisms	As Pharmaceutical Products	As Food Additives	Qualified Presumption of Safety Microorganisms
*Lactobacillus acidophilus*	+		+
*Lactobacillus amylovorus*		+	+
*Lactobacillus casei*	+	+	+
*Lactobacillus gasseri*	+		+
*Lactobacillus helveticus*	+		+
*Lactobacillus johnsonii*		+	+
*Lactobacillus pentosus*		+	+
*Lactobacillus plantarum*		+	+
*Lactobacillus reuteri*	+		+
*Lactobacillus rhamnosus*	+	+	+
*Bifidobacterium adolescentis*	+		
*Bifidobacterium animalis*	+		+
*Bifidobacterium bifidum*	+		
*Bifidobacterium breve*		+	
*Bifidobacterium infantis*	+		
*Bifidobacterium longum*	+		+
*Enterococcus faecium*	+		
*Lactococcus lactis*		+	+
*Streptococcus thermophilus*	+		+
*Bacillus clausii*	+		+
*Escherichia coli Nissle* 1917	+		
*Saccharomyces cerevisiae* (*boulardii*)	+		+

+: used as.

**Table 3:** Clinical studies and the effect of probiotic administration on human health.

Probiotic Microorganisms	Reported Specific Benefits in Indicated References	References
**Overweight and Obesity**		
*Enterococcus faecium, Streptococcus thermophilus*	Reduction in body weight, systolic Blood Pressure LDL-C (Low-Density Lipoprotein Cholesterol) and increase in fibrinogen levels.	[63]
*Lactobacillus gasseri* SBT2055	Significant decrease in body mass index (BMI), waist, abdominal Visceral Fat Area (VFA) and hip circumference.	[64]
*Lactobacillus salivarius* Ls-33	Increase in the ratios of *Bacteroides*, *Prevotellae* and *Porphyromonas*.	[65]
*Lactobacillus gasseri* SBT2055	Decrease in BMI and arterial blood pressure values.	[66]
*Lactobacillus plantarum*	Reduction in BMI and arterial blood pressure levels.	[67]
*Lactobacillus acidophilus* La5, *Bifidobacterium lactis* Bb12, *Lactobacillus casei* DN001	Drastic modifications in gene expression in PBMCs as well as BMI, fat percentage and leptin values.	[68,69,70]
*Bifidobacterium, Streptococcus thermophilus*	Improvement in lipid profile, insulin sensitivity, and decrease in CRP (C-reactive protein).	[71]
*Lactobacillus paracasei* N19	No effects have been noticed.	[72]
*Lactobacillus acidophilus* La5, *Bifidobacterium animalis* Bb12	Significant drop in fasting glucose concentration and increase in HOMA-IR (Homeostasis Model Assessment of Insulin Resistance).	[73]
**Type-2 diabetes and Dyslipidemia**		
*Lactobacillus acidophilus* La5, *Bifidobacterium lactis* Bb12	Total cholesterol (TC) and LDL-C improvement.	[74]
*Lactobacillus acidophilus* La5, *Bifidobacterium lactis* Bb12	Decreased fasting blood glucose and antioxidant status.	[75]
*Bifidobacterium animalis* DSMZ 23733, *Bifidobacterium breve* DSMZ 23732	Reduction of total cholesterol (TC).	[76]
*Lactobacillus acidophilus* La-5, *Bifidobacterium animalis* BB-12	Improved HDL-C levels and reduced LDL-C/HDL-C ratio.	[77]
*Lactobacillus plantarum* A7	Decreased methylation process, SOD (superoxide dismutase).	[78]
*Lactobacillus acidophilus* La-5, *Lactobacillus animalis* BB-12	Significant difference between groups concerning mean changes of HbA1c, TC, and LDL-C.	[79]
*Lactobacillus acidophilus, Lactobacillus reuteri* NCIMB	Reduced LDL-C (Low-Density Lipoprotein Cholesterol) levels.	[80]
*Lactobacillus acidophilus*	A significant reduction was found in LDL.	[81]
*Lactobacillus reuteri* NCIMB 30242	Reduced low-density lipoprotein cholesterol by 11.64% and total cholesterol by 9.14% in hypercholesterolemic adults	[82]
Various strains of LAB	Control of blood cholesterol-Hypocholesterolemia-effect and hyperlipidemia	[83]
*Weissella koreensis*	Significant anti-obesity effect	[84]
**Constipation**		
*Bifidobacterium animalis* DN-173 010, *Escherichia coli* Nissle 1917 *Lactobacillus casei* Lcr35	Treatment of functional constipation in adults.	[85]
*Bifidobacterium lactis*	Improvement of the whole gut transit time, stool frequency, and stool consistency.	[86]
*Bifidobacterium animalis* subsp. *lactis*, BB-12(R)	Manage symptoms of occasional constipation	[87]
**Antibiotic-Associated Diarrhea, Diarrheas, Colic, Ulcerative colitis**		
*Saccharomyces cerevisiae*, *Saccharomyces boulardii*	Reduction of diarrhea rates in children receiving probiotic yeast (7.5%) compared to those receiving placebo (23%).	[88]
*Lactobacillus reuteri* ATCC 55730	Elimination of pain and symptoms in direct association with intestinal colic.	[89]
Probiotic VSL#3	Remission in 42.9% of patients in the probiotic group versus 15.7% in the placebo group.	[90]
*Escherichia coli* Nissle 1917	Treatment of inflammatory bowel disease.	[91]
*Bifidobacterium longum* CMCC P0001	Treatment of gastro-intestinal disorders.	[92]
*Lactobacillus, Bifidobacterium*	Reduction of the incidence of severe necrotizing enterocolitis by 57% and the risk of mortality by 35%.	[93]
*Lactobacillus rhamnosus, Saccharomyces boulardii*	A protective role in preventing antibiotic-associated diarrhea after intake of 50^11^ CFU/day.	[94]
*Lactobacillus* GG	Probiotics may decrease duration of acute diarrhoea in infants and children by ~1 day	[95]
*Bacillus licheniformis*	Reduce the effect of antibiotics use in treatment of diarrhea and can detoxify aflatoxin B1up to 94.7% in food matrixes.	[96]
*Bacillus clausii*	Treatment of acute diarrhea in children	[97]
*Lactobacillus reuteri*	Reduced crying time by an average of 25.4 min per day and Treat colic in breastfed infants	[98]
*Bacillus clausii* UBBC-07	Reduced severity of diarrhea in children under 5 years of age	[99]
**Alleviation of lactose intolerance**		
*Streptococcus lactis, Streptococcus plantarum, Streptococcus cremoris, Streptococcus casei, Streptococcus diacetylactis, Streptococcus florentinus, Streptococcus cremoris*	Improved lactose digestion and tolerance.	[100]
*Lactobacillus delbrueckii subsp. bulgaricus* and *Streptococcus thermophilus*	Consumption of live yogurt cultures in yogurt improves the digestion of lactose present in yogurt in individuals with lactose maldigestion. Yogurt should contain at least 10^8^ CFU live probiotic strains per gram	[101]
*Bifidobacterium animalis* DSM 26137 and *Lactobacillus plantarum* DSM 26329	Significant reduction of diarrhea frequency and flatulence.	[102]
**Allergic Rhinitis**		
*Streptococcus paracasei-33*	Clinical improvements in nasal blockage, rhinorrhea, and nasal itching.	[103]
*Lactobacillus paracasei*-33	Significant evidence of beneficial clinical and immunologic effects of probiotics in the treatment of seasonal Allergic Rhinitis.	[104]
**Blood Pressure**		
Various strains of *Lactobacillus* sp.	Regulation of blood pressure.	[105]
*Lactobacillus helveticus* and *Saccharomyces cerevisiae*	Reduction of hypertension effects	[106]
**Atopic Dermatitis**		
*Lactobacillus fermentum* VRI 033 PCC™	Reduction in SCORAD (SCORing Atopic Dermatitis).	[107]
*Bifidobacterium animalis* subsp *lactis*	Important decrease in the sternness of atopic dermatitis with an improvement in the ration of IFN- and IL-10.	[108]
*Lactobacillus rhamnosus* HN001	Substantially reduced the cumulative prevalence of eczema in infants	[109]
**Cancer and side effects associated with cancer**		
*Lactobacillus rhamnosus* 573	Patients had less abdominal discomfort, with less hospital care and fewer chemo dose reductions.	[110]
*Lactobacillus acidophilus, Bifidobacterium bifidum*	Reduction in incidence of diarrhea and better stool consistency.	[111]
*Lactobacillus plantarum* CGMCC 1258, *Lactobacillus acidophilus* LA-11, *Bifidobacterium longum* BL-88	Significant improvement in the integrity of gut mucosal barrier and reduction in infections complications.	[112]
*Lactobacillus casei* Shirota (LcS)	Significant evidence of cancer preventing particularly colorectal cancer.	[113]
*Lactobacillus casei* ATCC 393	Significant in vivo anti-proliferative effects accompanied by apoptotic cell death in colon carcinoma cells.	[114]
*Lactobacillus acidophilus* and *Bifidobacterium* spp.	Inhibit growth of tumor cell, produce anti-carcinogens and reduces cancer risks	[115]
*Lactobacillus paracasei*	Anticancer activity	[116]
**Bacterial Vaginosis**		
*Lactobacillus rhamnosus*	The vaginal administration of the probiotic strain leads to stabilization of the vaginal flora with obvious reduction of bacterial vaginosis recurrence.	[117]
*Lactobacillus gasseri* LN40, *Lactobacillus fermentum* LN99, *Lactobacillus casei* LN113, *Pediococcus acidilactici* LN23	Strain LN is characterized by a high colonial rate in the vagina bacterial vaginosis, patients and women receiving LN strain were totally cured 2–3 days after administration.	[118]
*Lactobacillus acidophilus* La-14^®^ and *Lactobacillus rhamnosus* HN001^®^	The addition of a combination of the probiotic strains La-14^®^ and HN001^®^ alongside bovine lactoferrin to antibiotic treatment, was shown to significantly improve symptoms of BV. It also decreased the recurrence rate, as compared with antibiotic treatment alone.	[119]
*Lactobacillus crispatus* CTV-05	The administration of 2 billion CFU of *L. crispatus* CTV-05 to 228 premenopausal women with recurrent BV using a vaginal applicator daily for 24 weeks led to 30% of recurrence of BV in the intervention group compared with 45% of the placebo group	[120]
**Depression, Anxiety and Mental disorders**		
*Lactobacillus helveticus* R0052 *Bifidobacterium longum* R0175	Probiotic supplementation reduced aggressive and ruminative thoughts in response to sad mood.	[121]
*Lactobacillus, Bifidobacterium*	Beneficial effects on mental health and mood.	[33]

**Table 4:** Clinical studies and reported specific benefits to human health—Microbial antagonism.

Probiotic Microorganisms	Main Results—Microbial Antagonism	References
**Antifungal activity**		
*Lactobacillus acidophilus* ATCC 4495, *Lactobacillus plantarum* NRRL B-4496	Significant antifungal activity.	[135]
*Lactobacillus acidophilus, Bifidobacterium lactis, Bifidobacterium longum, Bifidobacterium bifidum*	Probiotic strains have the potential to reduce enteral fungal colonization and decrease invasive fungal sepsis rates in low–birth-weight neonates.	[136]
*Lactobacillus acidophilus* ATCC 4356	*L. acidophilus* produced substances with anti-*Candida* activity, reducing its growth by 45.1%.	[137]
*Lactobacillus buchneri*	Antagonistic potential against *Candida albicans*	[131]
**Eradication of *Helicobacter***		
*Lactobacillus casei* Shirota	Inhibition of the growth of *Helicobacter pylori* (by 64% in the probiotic group and by 33% in the control).	[138]
*Pediococcus acidilactici* BA28	Significant rates of elimination of *H. pylori* infections.	[139]
*Lactobacillus plantarum* ZDY 2013	Preventive effects against *H. pylori.*	[140]
*Lactobacillus acidophilus, Bifidobacterium animalis*	A significant efficacy in *H. pylori* eradication.	[141]
*Lactobacillus fermentum* UCO-979C	Inhibition of the function of *H. pylori* by regulating the immune system.	[142]
**Antimicrobial activity**		
*Lactobacillus acidophillus*	Antimicrobial activity against *Campylobacter jejuni* and *Listeria monocytogenes*	[143]
*Lactobacillus casei*	Antagonistic potential against *Cronobacter sakazakii*, *Cl. jejuni* and *L. monocytogenes*	[143]
*Lactobacillus plantarum*	Microbial antagonism against *Salmonella enteritidis*, *Cr. sakazakii*, *Cl. jejuni*, *L. monocytogenes* and *E. coli*	[143]
*Lactobacillus lactis*	Antimicrobial activity against *S. enteritidis*, *Cr. sakazakii*, *Cl. jejuni*, *L. monocytogenes* and *E. coli*	[143]
*Bifidobacterium bifidum*	Antagonistic activity against *Cr. sakazakii*, *Cl. jejuni*, *L. monocytogenes* and *E. coli*	[143]
*Lactobacillus salivarius*	Antimicrobial activity against *L. monocytogenes*, *S. enteritidis*, *St. mutans*, *Candida albicans*, *Cr. sakazakii* and *Cl. Jejuni*	[143,144]
*Lactobacillus rhamnosus*	Microbial antagonism against *S. enteritidis*, *Cr. sakazakii*, *Cl. jejuni*, *L. monocytogenes*, *E. coli* and *Clostridium difficile*	[143,145]
*Weissella cibaria* and *Weissella koreensis*	Antimicrobial activity against *L. monocytogenes*, *E. coli* and *Salmonella* spp.	[84]
LAB	Effective against *Salmonella enterica* ver. *Typhimurium*, Rota viral infections and *Clostridium difficile* diarrhea	[145]
*Lactobacillus paracasei*	*E. coli* and *Listeria innocua* inhibition effects	[116]
